# Individualized and Platform-Designated Gene-Editing Studies in the Registered Clinical Record: A Case-by-Case Census Against 148 Eligible Studies

**DOI:** 10.7759/cureus.117975

**Published:** 2026-10-15

**Authors:** Oscar Mingtian Li, Yijiang Li

**Affiliations:** 1 Zweistein Innovation Lab, Active Care and Education in Sports and Outreach (ACESO) Foundation, Dublin, USA

**Keywords:** clinical trial registries, crispr, expanded access, gene editing, n-of-1 trials, platform technology designation, study design

## Abstract

Introduction

In February 2025, a single infant with carbamoyl phosphate synthetase 1 deficiency received a customized base-editing therapy, and individualized gene editing has since been discussed as an emerging category of care. No one has yet counted whether this category exists in the registered study record.

Methods

We conducted a cross-sectional census of interventional studies of human gene-editing agents registered on ClinicalTrials.gov. Records were retrieved through the ClinicalTrials.gov application programming interface, version 2, with a verbatim, intervention-scoped query and a fixed census date of 17 September 2026. Eligibility was verified from each record's own fields, and every exclusion is supported by the field value that justifies it. Two independent screening passes classified each eligible record as single-participant or n-of-1, multi-variant umbrella or platform-designated, or conventional cohort. Agreement was quantified with Cohen's kappa and a 95% confidence interval (CI). Records in the individualized and platform classes were re-verified against their complete registry entries and version histories. We also searched public US Food and Drug Administration (FDA) records for the regulatory route of each census record. Reporting follows the Strengthening the Reporting of Observational Studies in Epidemiology (STROBE) statement.

Results

Of 212 retrieved records, 64 were excluded, and 148 were eligible. Four eligible records were single-participant or n-of-1 designs, and three were multi-variant umbrella or platform-designated designs, giving a census of seven studies. Of the remainder, 140 were conventional cohorts, and one was an editing arm inside a non-editing platform trial. Version histories confirmed the four single-participant designs; one of them reached an enrolment of one through a revision made before enrolment began. The same histories showed that three other records had an enrolment of one or two only because of termination or under-enrolment. Only two of the 148 eligible records describe a construct designed for the individual participant. No publicly verifiable platform technology designation was located for any census record, and the census query returned no expanded-access record. Of 23 studies completed more than 12 months before the census, five had posted results (21.7%, Wilson 95% CI: 9.7%-41.9%); because this interval is wide, the figure is reported as context only. Agreement on design class was moderate (kappa, 0.540; 95% CI, 0.264-0.815).

Conclusions

Nineteen months after the first individualized CRISPR treatment, the registry contains seven individualized or platform-designated gene-editing studies, and only two records describe an individualized construct. The regulatory pathway used by the index case was not retrievable from the registry, and no regulatory route could be verified from public records; this is an absence of public evidence, not evidence that no route existed. These counts describe the ClinicalTrials.gov record, not the prevalence of individualized gene editing, and the design-class counts are provisional given moderate agreement.

## Introduction

In February 2025, an infant with severe carbamoyl phosphate synthetase 1 deficiency received a base-editing therapy designed for his specific variant. He was dosed at approximately seven and eight months of age under a single-patient expanded-access Investigational New Drug application [1]. The clinical path to that point began with zinc finger nuclease-edited autologous T cells and transcription activator-like effector nuclease (TALEN)-edited allogeneic chimeric antigen receptor T cells [2,3]. It continued through ex vivo CRISPR-Cas9 editing for sickle cell disease and beta-thalassemia and in vivo editing in the liver and the retina [4-7]. Meanwhile, a patient-customized antisense oligonucleotide had already shown that a drug can be designed for one person and delivered under a single-patient regulatory pathway [8]. The index report was widely read as the start of individualized gene-editing care, and subsequent commentary has treated bespoke editing as a category rather than an exception [9,10]. This expectation has a real technical basis. Base and prime editors are programmable, and their delivery chemistry is reusable, and the authors of the index case argued that the platform nature of the technology makes corrective editing well suited to rapid customization for individual patients [1,11,12].

How a field describes itself and what it registers are different things. Trial registration is where a study declares its design, enrolment unit, and intervention in a structured, public, and dated form. It is also the only source in which planned studies, rather than published ones, can be counted. If individualized editing is becoming an established practice, the registry is where this shift should appear first: as single-participant designs, as master protocols that manufacture a product for each participant, or as platform designations attached to a reusable technology.

Two recent analyses describe the gene-editing trial landscape, but neither answers this question. A registry-wide analysis of CRISPR therapeutics enumerated trials on ClinicalTrials.gov and the EU Clinical Trials Register and described their distribution by indication, editing modality, and sponsor [13]. It did not classify records by design class, verify eligibility from each record's own fields, or count individualized or platform designs. A scoping review identified 29 published gene-editing trials up to May 2025 and examined design features that might accelerate clinical adoption, including editing technology, target cells, indications, phase, and delivery [14]. Its unit of analysis was the published trial report rather than the registered record, and it did not count individualized or platform-designated designs either. The present study differs in three respects: Its unit is the registered record rather than the publication; eligibility is verified field by field, with duplicate screening and a reliability statistic; and its primary outcome is a census by design class.

The regulatory framework matters for the same reason. The Food and Drug Omnibus Reform Act created a platform technology designation that lets a sponsor carry data about a reusable technology across products. However, the statute makes a platform eligible only if it is already incorporated in or used by an approved or licensed drug [15,16]. Individualized editing fits poorly into this structure because each product is used only once. How the few individualized and platform-designated editing studies reached patients, and which regulatory instrument each used, is therefore part of the description rather than a separate question.

The primary objective was to identify and characterize every individualized or platform-designated gene-editing study registered on ClinicalTrials.gov at a fixed census date, described case by case against a denominator of all eligible registered gene-editing studies. There were three secondary objectives. The first was to classify every eligible record by design class and to measure how reproducibly that classification can be made from registry fields. The second was to identify the records whose own text describes a construct designed for the individual participant. The third was to determine whether the regulatory route of each census study can be verified from public sources.

## Materials and methods

Design and reporting

This is a cross-sectional census of registered study records at a fixed census date, reported according to the Strengthening the Reporting of Observational Studies in Epidemiology (STROBE) statement [17]. The Preferred Reporting Items for Systematic Reviews and Meta-Analyses (PRISMA) statement does not apply because the unit of analysis is a registered study record retrieved from a structured database rather than a published report found through a bibliographic search, and no study results are synthesized. We nevertheless provide a PRISMA-style flow diagram at the start of the Results, because the screening path has the same shape and readers may wish to audit it.

Data source and search

Records were retrieved from the /studies endpoint of the ClinicalTrials.gov application programming interface version 2 [18]. The query was restricted to the intervention field (query.intr), and the verbatim query string was as follows: CRISPR OR Cas9 OR Cas12 OR "base editing" OR "prime editing" OR "zinc finger nuclease" OR ZFN OR TALEN OR "genome editing" OR "gene editing".

The census date was 17 September 2026. The interface reported 212 matching records, and all 212 were retrieved. We deliberately did not filter the query by study type so that exclusions for non-interventional designs would be made from each record rather than by the search. For reference, the same terms searched across all record fields return 285 records. The 73 additional records mention editing vocabulary somewhere other than the intervention field and fall outside the scope of this instrument.

Eligibility

A record was eligible if it registered an interventional study that administered a gene-editing agent to human participants. Eligibility was verified from the record's own fields and was never assumed from the query match. Exclusions were counted under five reasons, each supported by the field value that justified it: non-interventional by the StudyType field, laboratory assay only, gene addition or transfer with no editing component, withdrawn with no enrolment, and no gene-editing intervention for another stated reason. The fifth reason was not among the four specified at the outset; it was added because it was unavoidable. Several interventional records match the editing vocabulary but administer no editing agent to a human genome. Examples include a product whose record states that it does not edit or permanently change the gene, a CRISPR-engineered malaria parasite, and gene-edited food. Reasons were applied in a fixed priority order (non-interventional, laboratory assay only, gene addition or transfer, and then withdrawn with no enrolment) so that a withdrawn observational record is counted under study type.

Screening and design classification

Every retrieved record went through two independent screening passes. The first applied deterministic field rules, together with a locator for editing vocabulary. For each match, the locator recorded the field path, the matched term, and the surrounding text so that the location of the evidence can be audited. The second pass was an independent record-by-record reading of the same fields, blinded to the first. It had to name the field on which each decision rested and quote it verbatim.

The second pass was performed by a large language model, Claude Sonnet 5 (Anthropic, San Francisco, CA), accessed through the Claude Science interface on 17 September 2026 at the provider's default sampling temperature. The log records the model identifier but no finer build string. For each record, the model received a fixed template of labelled registry fields and nothing else: National Clinical Trial (NCT) identifier; study type; overall status with the first 200 characters of the whyStopped field; enrolment count and type; phases; primary completion date and type; lead sponsor; brief and official titles; conditions and keywords (300 characters each); arm groups with their count and labels (300 characters); the type, name, other names, and description of each intervention (400 characters each); brief summary (2,500 characters); and detailed description (1,800 characters). The system instruction, reproduced verbatim in the Appendices, required the model to decide from the supplied fields only and to return a structured response giving eligibility, exclusion reason, evidence field and verbatim quote, design class with its evidence, and an ambiguity flag. All 212 records were submitted in one batch, and 18 responses that were cut off before the structured output was complete were re-requested with a larger output limit. The 212 per-record inputs are available from the corresponding author.

Agreement was quantified with Cohen's kappa [19] and described using the Landis and Koch scale [20]. Agreement on eligibility was kappa 0.718 (95% confidence interval {CI}, 0.618-0.817; 87.3% agreement, n = 212). All 27 disagreements were adjudicated against each record's own field text. Two further records were overturned after both passes had agreed, following a check for misclassification in both directions.

Each eligible record was then classified as single-participant or n-of-1, multi-variant umbrella or platform-designated, or conventional cohort. A single-participant design was defined as a registered enrolment unit of one participant. A multi-variant umbrella or platform-designated design was defined as a single master protocol spanning multiple variants, diseases, or sub-studies or as a record that describes itself as a platform, basket, or master protocol. Dose-escalation cohorts of one product in one indication were not counted as platform designs. A conventional cohort was any other eligible record, that is, a fixed cohort receiving one defined product. A classification was flagged as ambiguous when the fields of the record supported more than one class or were too sparse to support a choice. The working definitions supplied to the second pass are reproduced in the Appendices; their wording of the single-participant class is broader, and the definitions stated here are the ones applied at adjudication and in every reported count.

Tie-break rule for overlapping classes

Two records met both the umbrella and the single-participant definitions, because a master protocol can manufacture a bespoke product for each participant. The tie-break rule was fixed before any class counts were seen and was not changed afterward. Classification follows the unit that the protocol holds constant: A record whose protocol keeps one product fixed and varies the population is a platform or umbrella design, and the single-participant class is reserved for records whose registered enrolment unit is one participant. A separate, non-exclusive yes/no attribute records whether the record's own text describes a construct designed for the individual participant; this attribute was assigned to all 148 eligible records from their own text. The rule keeps the primary classes mutually exclusive, which kappa requires, while still allowing individualization to be counted.

One eligible record registers a construct made for each participant but uses no umbrella, platform, or master-protocol language. Read literally, the product-variation clause of the rule would move this record into the platform class. Read as a tie-break that applies only to records meeting both definitions, it would leave the record as a conventional cohort that carries the individualization attribute. We used the narrow reading as primary and report the literal reading as a sensitivity analysis.

Reverification of census records

Every record in the individualized and platform classes was re-verified against its complete registry entry, rather than the flattened fields, and against its version history. The version history shows the enrolment as originally registered and the overall status at that time. This allows a study designed for one participant to be distinguished from a study whose enrolment fell to one because of termination or under-enrolment, which the current fields alone cannot do.

Both passes classified one record as platform-designated: a 20-arm platform trial in rare solid tumors in which exactly one arm uses a gene-editing agent. We removed it from the platform class because a single editing arm cannot show how gene editing is organized but kept it in the eligible corpus and described it separately. Per-record verification also exposed a related discrepancy. The same editing product has its own separate registration, which describes it only as a genetically modified natural killer cell product without stating an editing mechanism. That record was therefore excluded for the lack of editing evidence in its own fields, even though the platform record describes the product as base-edited.

Regulatory-route layer

We searched public sources for the regulatory route of each census record: the openFDA Drugs@FDA and structured product labelling endpoints, the statutory text and US Food and Drug Administration (FDA) guidance on the platform technology designation program, the registry's own expanded-access records and oversight fields, and the published report of the index case [1,15,16,21,22]. No route could be verified from public sources for any census record, for reasons set out in the Results, so this layer is reported as an absence of publicly verifiable evidence and not as a table of routes. An absence of public evidence is not treated as evidence that no regulatory instrument was used.

Result record

For studies with an overall status of completed and a primary completion date of more than 12 months before the census, we tabulated the registry's result-posted flag and calculated binomial proportions with Wilson score 95% confidence intervals [23]. This is descriptive context for a small subgroup of completed studies, not an outcome of this study, and we make no claim about compliance [24,25].

Novelty assessment

We searched PubMed through the National Center for Biotechnology Information (NCBI) E-utilities (Bethesda, MD) for any earlier registry-based count of individualized or platform gene-editing studies and for any audit of result reporting in gene editing. Because the first search missed a relevant scoping review, we added a broader search for design-focused reviews of gene-editing trials. The counts are reported in the Results.

Statistics, data handling, and ethics

Analyses used Python 3.11 (Python Software Foundation, Beaverton, OR), with the pandas library (pandas development team) for data handling. The pandas version used at run time was not recorded; the same environment held pandas 2.3.3 when checked at resubmission. Figures were rendered with Matplotlib 3.10.9 (Matplotlib Development Team), the version recorded in the figure files' metadata. The retrieval, statistical analysis, and plotting code were written with Claude Opus 5 (Anthropic, San Francisco, CA) and subsequently reviewed and verified by the first author. Cohen's kappa and Wilson score intervals were computed with hand-written closed-form code rather than a statistical library. The kappa confidence interval used the simplified large-sample standard error:

$$SE(\kappa) = \sqrt{\frac{p_o(1 - p_o)}{n(1 - p_e)^2}}$$

where po is the observed agreement, pe is the agreement expected by chance, and n is the number of records. Wilson intervals used z = 1.96. When recomputed with the Fleiss-Cohen-Everitt asymptotic standard error, the design-class interval is 0.300-0.779, and the eligibility interval is 0.619-0.816, so the simplified intervals reported here are not narrower than the conventional ones. No values were imputed. No record was dropped for missing data: every retrieved record has a decision, and fields that a record does not state are reported as not stated rather than inferred. The study used only publicly available, de-identified registry and regulatory records and involved no human participants or identifiable private information; it was therefore exempt from institutional review board review.

## Results

The census

Of 212 retrieved records, 64 were excluded at screening, and 148 were eligible (Figure 1). The reasons for exclusion were non-interventional design (25), gene addition or transfer with no editing component (12), withdrawal with no enrolment (10), laboratory assay only (nine), and no gene-editing intervention for another stated reason (eight).

**Figure 1:**
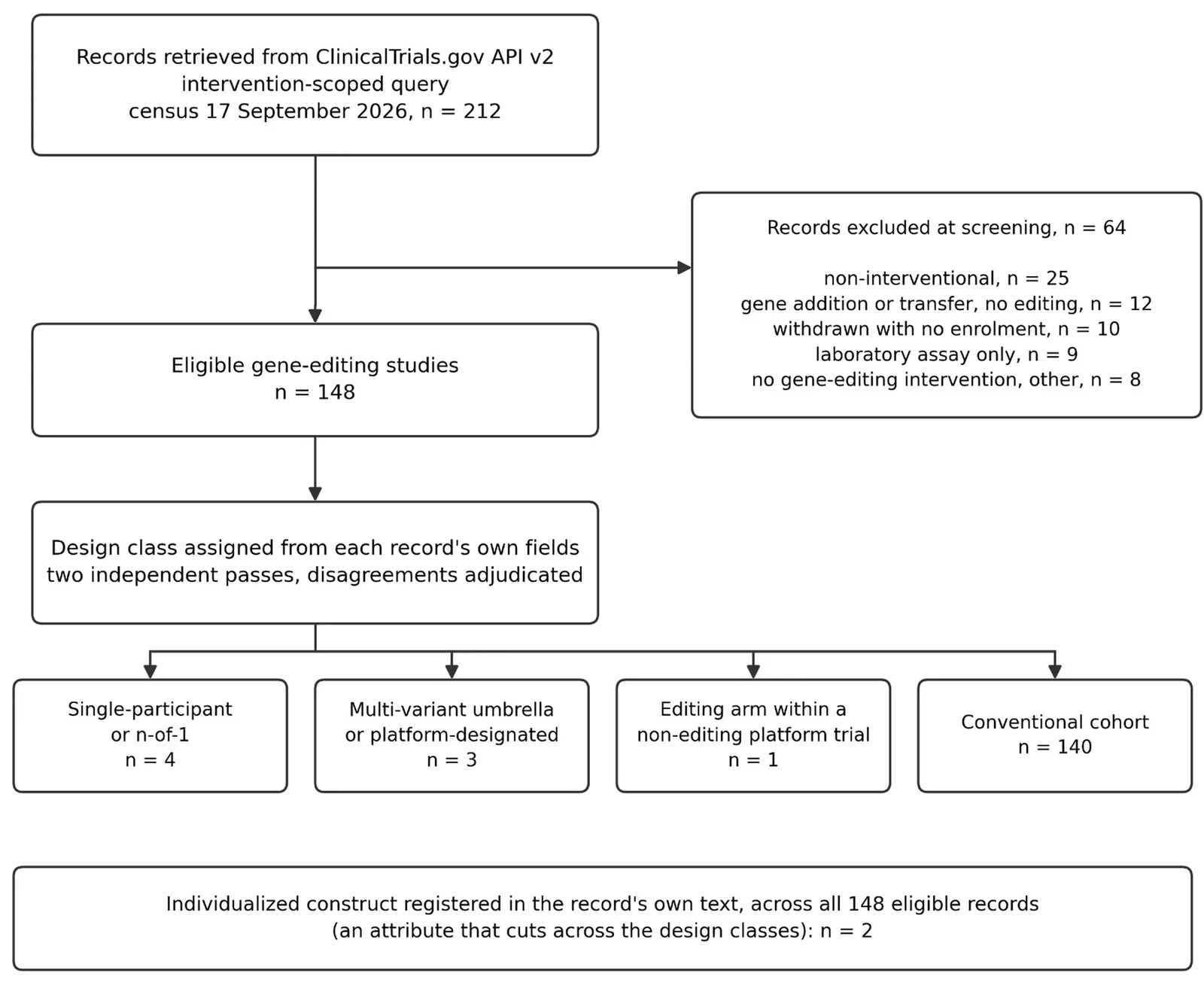
Flow of records from retrieval to design classification Records were retrieved from the ClinicalTrials.gov application programming interface version 2 with an intervention-scoped query, at a census date of 17 September 2026 [18]. Exclusion reasons are mutually exclusive and were applied in the priority order stated in the Methods so that a withdrawn observational record is counted under non-interventional. The individualized-construct attribute is non-exclusive and was assigned to all 148 eligible records from their own text; it is shown at the foot of the diagram because it cuts across the four design classes rather than subdividing one of them. The diagram follows the shape of a PRISMA flow for auditability; PRISMA itself does not apply to this design, as stated in the Methods API, application programming interface; PRISMA, Preferred Reporting Items for Systematic Reviews and Meta-Analyses

Of the 148 eligible studies, four were single-participant or n-of-1 designs, and three were multi-variant umbrella or platform-designated designs. These seven studies form the census, representing 4.7% of the eligible population. This proportion describes records registered on ClinicalTrials.gov at the census date and is not an estimate of how common individualized gene editing is in practice. A further 140 records were conventional cohorts, and one was an editing arm inside a non-editing platform trial. Table 1 lists the seven census records by registration number. Figure 2 shows when each was first registered, together with an eighth record. This eighth record is not a census study: it is a conventional cohort that describes a construct designed for the individual participant, plotted for comparison because it would enter the platform class under the sensitivity analysis described below.

**Figure 2:**
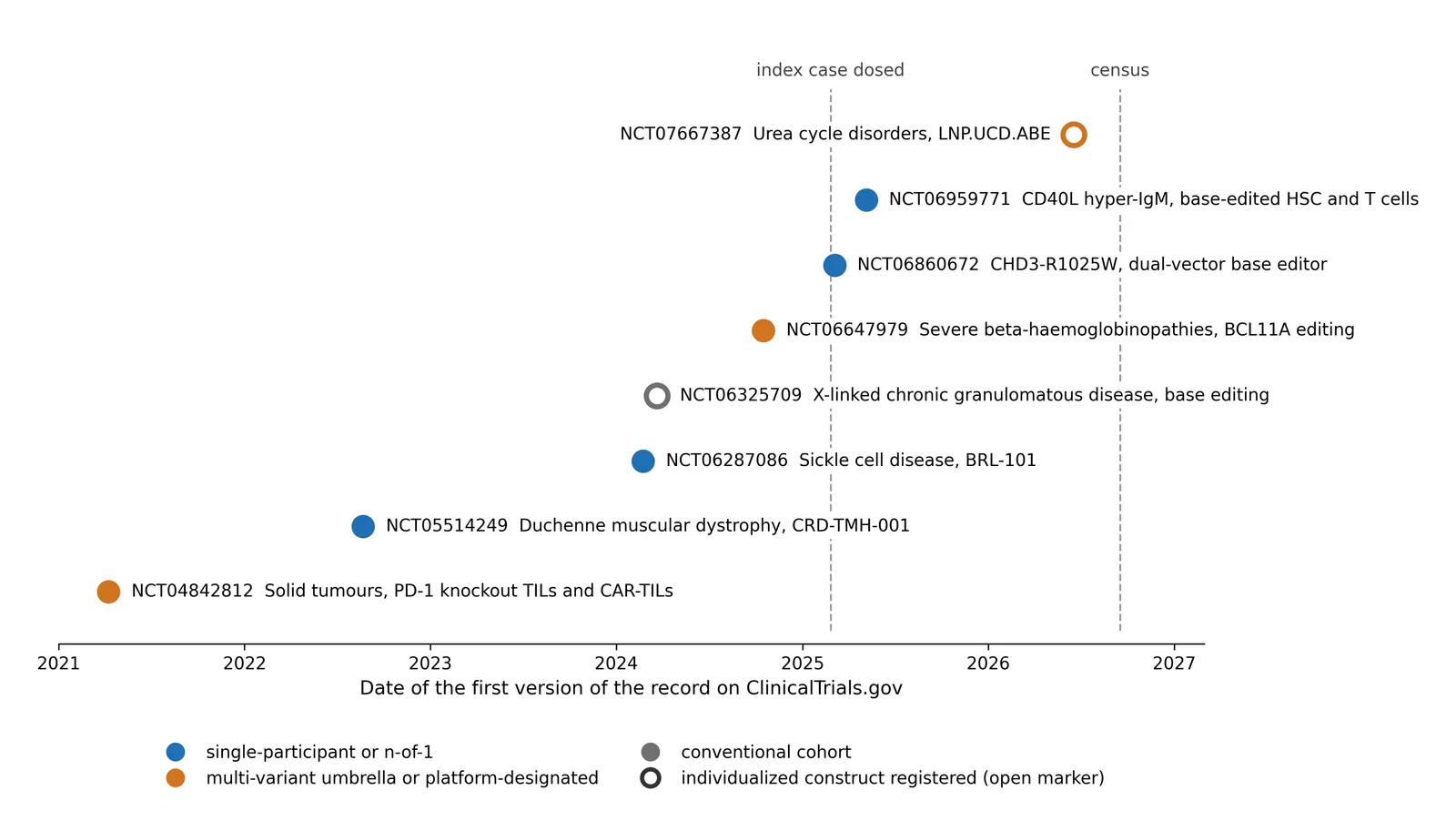
Date of the first registry version of the seven census records and of one individualized cohort record Each marker is one registered record, placed on the x-axis at the date of the first version of that record in the registry's version history. Marker color shows the design class assigned after adjudication, and an open marker indicates that the record's own text describes a construct designed for the individual participant. The figure shows the seven census records, NCT06959771 [26], NCT05514249 [27], NCT06860672 [28], NCT06287086 [29], NCT04842812 [30], NCT07667387 [31], and NCT06647979 [32], and one additional record, NCT06325709 [33]. This eighth record is a conventional cohort that describes a construct designed for the individual participant; it is not part of the census and would enter the platform class only under the sensitivity analysis described in the Results. The left dashed line marks 25 February 2025, when the index individualized base-editing therapy was first administered, and the right dashed line marks the census date, 17 September 2026 [1]. Three of the eight records shown were first registered after the index case was dosed. BRL-101 and CRD-TMH-001: sponsor product codes NCT, National Clinical Trial registration number; LNP, lipid nanoparticle; UCD, urea cycle disorder; ABE, adenine base editor; CD40L, CD40 ligand; HSC, hematopoietic stem cells; CHD3, chromodomain helicase DNA-binding protein 3; BCL11A, B-cell lymphoma/leukemia 11A; PD-1, programmed cell death protein 1; TILs, tumor-infiltrating lymphocytes; CAR, chimeric antigen receptor

**Table 1:** Individualized and platform-designated gene-editing studies registered on ClinicalTrials.gov at the census date, keyed by registration number Design class was assigned from each record's own fields by two independent passes, with disagreements adjudicated; the tie-break rule for records meeting more than one class definition is stated in the Methods. The date of the first registry version and the enrolment at that version are taken from version 0 of each record's version history. Enrolment at census is taken from the record as retrieved on 17 September 2026. Both enrolment values come from the registry's enrolment field [18]. Dates are given as yyyy-mm-dd. The number in square brackets after each NCT number is the reference number of that trial's ClinicalTrials.gov record NCT: National Clinical Trial registration number

NCT number	Design class	Condition	Lead sponsor	Registered phase	Date of first registry version (yyyy-mm-dd)	Enrolment at first registry version	Enrolment at census	Enrolment type at census	Overall status at census
NCT06959771 [26]	Single-participant/n-of-1	CD40 ligand hyper-IgM syndrome	National Institute of Allergy and Infectious Diseases (NIAID)	Phases 1 and 2	2025-05-06	1	1	Estimated	Recruiting
NCT05514249 [27]	Single-participant/n-of-1	Duchenne muscular dystrophy	Cure Rare Disease, Inc.	Phase 1	2022-08-22	1	1	Estimated	Unknown
NCT06860672 [28]	Single-participant/n-of-1	CHD3-R1025W developmental disorder	Yongguo Yu	Early phase 1	2025-03-05	1	1	Actual	Terminated
NCT06287086 [29]	Single-participant/n-of-1	Sickle cell disease	Bioray Laboratories	Not applicable	2024-02-23	5	1	Estimated	Unknown
NCT04842812 [30]	Multi-variant umbrella or platform-designated	Advanced solid tumors	Second Affiliated Hospital of Guangzhou Medical University	Phase 1	2021-04-09	40	40	Estimated	Recruiting
NCT07667387 [31]	Multi-variant umbrella or platform-designated	Urea cycle disorders	Rebecca Ahrens-Nicklas	Phases 1 and 2	2026-06-18	7	7	Estimated	Recruiting
NCT06647979 [32]	Multi-variant umbrella or platform-designated	Severe beta-hemoglobinopathies	Daniel Bauer	Phase 1	2024-10-16	10	10	Estimated	Recruiting

The four single-participant designs are as follows: NCT06959771 registers base-edited hematopoietic stem cell and T cell gene therapy for CD40 ligand hyper-IgM syndrome [26]. Its official title ends "Single Patient Study," and its enrolment was one at first posting in May 2025 and remained one at census across 54 record versions. NCT05514249, titled "Treatment of a Single Patient With CRD-TMH-001," concerns a rare Duchenne muscular dystrophy variant [27]; its arm group is labelled "single patient," and its enrolment was one both at first posting and at census. NCT06860672 registers a dual-vector adeno-associated virus base editor for the CHD3-R1025W variant [28]. Its estimated enrolment of one was set at first posting in March 2025, while the record was recruiting and was therefore planned before any outcome occurred. At census, the record was terminated with an actual enrolment of one, and the whyStopped field states that the single participant died. NCT06287086 became a single-participant design through a revision rather than at original registration: Its enrolment was reduced from five to one at record version 2 in March 2024, while the study was not yet recruiting, and it remained one at census [29].

The three platform or umbrella designs are as follows: NCT07667387 is an official "master protocol" for an umbrella trial in urea cycle disorders [31]. In the record's own words, each subject will have a personalized, variant-specific lipid-nanoparticle adenine base editor developed and evaluated during a screening period of up to eight months; the registered enrolment is seven. NCT06647979 is registered as an adaptive basket trial that applies one editing approach to both sickle cell disease and transfusion-dependent beta-thalassemia, with an enrolment of 10 [32]. NCT04842812 registers several engineered tumor-infiltrating lymphocyte and chimeric antigen receptor constructs with programmed cell death protein 1 (PD-1) knockout under a single arm, with an enrolment of 40 [30]. The editing step is common to every construct, which distinguishes this record from the platform trial removed from this class (described below).

Enrolment of one is not the same as a single-patient design

Three further records have an enrolment of one or two at census but are not single-participant designs, and only the version history can show this. NCT02695160 registered nine participants, and its enrolment fell to one when it was terminated [34]. NCT07170254 registered 12 and fell to two, also on termination [35]. NCT06065189 registered five, fell to two, and is completed [36]. Counting current enrolment alone would have inflated the single-participant class from four to seven.

Individualized constructs are rarer than single-participant designs

Only two of the 148 eligible records describe a construct designed for the individual participant. One is the urea cycle master protocol described above [31]. The other, NCT06325709, is a National Institute of Allergy and Infectious Diseases study of base editing to repair mutations in hematopoietic stem and progenitor cells in X-linked chronic granulomatous disease, with 15 participants in a single arm [33]. Its detailed description states that each participant will undergo apheresis to collect CD34+ cells for the development and validation of a mutation-specific base-editing system. Under the narrow reading of the tie-break rule, it remains a conventional cohort that carries the individualization attribute. None of the four single-participant records uses personalization language anywhere; they are one-patient studies of products that are not described as bespoke.

In the sensitivity analysis using the literal reading of the tie-break rule, where a record that varies its product for each participant enters the platform class, the platform or umbrella count rises from three to four. If the 20-arm platform trial had also been kept in the class, the count would be five.

An editing arm inside a non-editing platform trial

NCT07307053 registers a platform trial in rare solid tumors with 20 arm groups and a planned enrolment of 600 [37]. Only one arm, a base-edited allogeneic natural killer cell product with a TIGIT knockout, uses a gene-editing agent. The record's individualization language refers to a different arm, an in vivo individualized T cell receptor therapy that involves no editing. The record is retained in the eligible corpus and reported separately, rather than being treated as evidence of how gene editing is organized. The same natural killer cell product is registered separately under NCT07083349, which describes it only as an allogeneic genetically modified natural killer cell product and states no editing mechanism [38]. That record was excluded because its own fields contain no editing evidence; the discrepancy between the two registrations of one product arises from verifying eligibility record by record.

Applying the classification is harder than applying the eligibility rule

Agreement on design class between the two independent passes was moderate: kappa 0.540 (95% CI: 0.264-0.815), with 93.2% raw agreement over 148 eligible records and 10 disagreements. By comparison, agreement on eligibility was kappa 0.718 (95% CI: 0.618-0.817). This gap reflects the registry rather than the raters. Eligibility depends on field values that a record either contains or does not, whereas design class depends on what a protocol intends, and intent is not a registered field. Fourteen of the 148 classifications were ambiguous on the record's own evidence, and the ambiguity followed recognizable patterns (Table 2): an individualized product inside a multi-patient protocol, where the fields do not say whether the design class is defined by the product or by the population; an enrolment of one or two that the current record cannot distinguish from early termination; rollover and long-term follow-up protocols whose design belongs to a parent study; and protocols that compare two editing platforms head to head. Given a moderate kappa with a confidence interval from 0.264 to 0.815, any single-rater count of design classes in this literature, including counts produced by automated classification, should be treated as provisional unless duplicate classification is reported.

**Table 2:** Eligible records whose own fields support more than one design class, keyed by registration number Each row gives the design class assigned after adjudication and the reason the record's fields are compatible with more than one class. EnrollmentCount, OverallStatus, briefSummary, and detailedDescription are ClinicalTrials.gov record fields. These records account for the moderate agreement on design class reported in the Results [19,20]. The number in square brackets after each NCT number is the reference number of that trial's ClinicalTrials.gov record NCT: National Clinical Trial registration number

NCT number	Assigned design class	Reason the fields support more than one class
NCT07804004 [39]	Conventional cohort	Multi-indication enrolment with one product; fields support conventional-cohort and umbrella readings
NCT03399448 [40]	Conventional cohort	Arms defined by disease indication, not by genetic variant
NCT04842812 [30]	Multi-variant umbrella or platform-designated	Multiple distinct constructs under one arm; fields do not state whether assignment is per-patient target
NCT05143307 [41]	Conventional cohort	Follow-up protocol; fields describe no design class of its own
NCT05795595 [42]	Conventional cohort	Multi-indication enrolment with one product
NCT07667387 [31]	Multi-variant umbrella or platform-designated	Master protocol across multiple urea cycle genes with per-subject personalized editors; fields support both umbrella and single-participant reading
NCT07307053 [37]	Editing arm within a non-editing platform trial	Record also uses "individualized"; platform protocol containing individualized arms
NCT03057912 [43]	Conventional cohort	Two distinct editing platforms compared inside one protocol
NCT06860672 [28]	Single-participant/n-of-1	EnrollmentCount=1 is ACTUAL under OverallStatus=TERMINATED; fields cannot separate single-patient design from early termination
NCT07170254 [35]	Conventional cohort	detailedDescription labels one arm "n=1"; fields support a one-participant arm inside a dose-finding cohort
NCT06287086 [29]	Single-participant/n-of-1	EnrollmentCount=1 but briefSummary/detailedDescription refers to plural subjects and sequential dosing
NCT04208529 [44]	Conventional cohort	Rollover protocol pooling participants from multiple parent trials
NCT06925542 [45]	Conventional cohort	Multi-indication enrolment with one product
NCT02695160 [34]	Conventional cohort	EnrollmentCount=1 (ACTUAL) in a three-cohort ascending-dose design; enrolment of one reflects termination, not a single-patient design

No regulatory route could be verified from public records

No platform technology designation was found in FDA public records for any census record (Table 3). This describes what public records can show and is not evidence that no designation was sought or granted. The FDA publishes no list of granted designations, so the only public evidence of a grant is an announcement by a sponsor or professional society. The single publicly documented grant, made in June 2025, is for an adeno-associated virus vector platform, which is not a gene-editing technology [22]. In addition, the statute makes a platform technology eligible only if it is already incorporated in or used by an approved or licensed drug [15,16,21]. Whether any product in this census relies on a technology that meets this condition cannot be established from public records.

**Table 3:** Regulatory markers for the census records, keyed by registration number The FDA-regulated drug field is the record's own oversight field value. Platform technology designation was sought in US Food and Drug Administration public records, which contain no roster of granted designations [15,16,22]. Expanded-access records were sought under the census query and among the oversight fields of all eligible records. Individualized construct records whether the record's own text describes a product designed for the individual participant. Sponsor country is derived from the record's location fields and is reported as not reported where those fields carry no country NCT, National Clinical Trial registration number; FDA, Food and Drug Administration

NCT number	FDA-regulated drug field	Expanded-access record	Platform technology designation in FDA public records	Individualized construct registered	Sponsor country
NCT06959771 [26]	Yes	No expanded-access record located	No public record located	No	United States
NCT05514249 [27]	Yes	No expanded-access record located	No public record located	No	United States
NCT06860672 [28]	No	No expanded-access record located	No public record located	No	China
NCT06287086 [29]	No	No expanded-access record located	No public record located	No	Not reported
NCT04842812 [30]	No	No expanded-access record located	No public record located	No	China
NCT07667387 [31]	Yes	No expanded-access record located	No public record located	Yes	United States
NCT06647979 [32]	Yes	No expanded-access record located	No public record located	No	United States

The census query returned no expanded-access record: No record had a study type of expanded access, and no eligible record had its expanded-access flag set to true. This absence matters for interpretation because the index case reached its patient by that route. According to the published report, the therapy was given as part of clinical care under a single-patient expanded-access Investigational New Drug application. The application was submitted when the patient was six months old and approved one week later, and the clinical protocol was reviewed by an institutional review board through alternative procedures, with FDA authorization for concurrence by the board's chairperson [1]. The route is therefore documented in the literature but was not retrievable from the registry by this census.

Some of this is documented, and some is not public. Documented are the statutory conditions for platform technology designation; the existence and date of one designation for a non-editing vector platform; and, from its primary report, the regulatory pathway, the timing of submission and approval, and the review mechanism of the index case. Not public are any list of designation applications or grants; the FDA correspondence for the index case, which is not held in a public docket and which we could not find in released form; and the regulatory status of the four census records whose oversight fields indicate an FDA-regulated drug, because an active investigational application is not a public record. Two of the seven census records are sponsored in China and are outside FDA jurisdiction. The openFDA Drugs@FDA and labelling endpoints returned no records for the approved editing product or for base-editing terms, because biologics licensed through the Center for Biologics Evaluation and Research are not included in those endpoints.

Completed studies and the result record as context

Twenty-three eligible studies had an overall status of completed and a primary completion date of more than 12 months before the census. All 23 dates were actual rather than estimated, and none was missing (Table 4). Of these, five had posted results in the registry (21.7%, Wilson 95% CI: 9.7%-41.9%). With so small a denominator, the interval spans more than a fourfold range and cannot support any claim about reporting behavior; the figure describes the completed subgroup and is not a finding. Across all 148 eligible records, 10 had posted results (6.8%, Wilson 95% CI: 3.7%-12.0%), a figure that includes studies not yet due to report.

**Table 4:** Eligible studies completed more than 12 months before the census, keyed by registration number Completion is the record's overall status field, and the date is the registry's primary completion date field, with its actual or estimated type given separately. Result posted is the registry's result-posted flag at the census date. These rows are descriptive context for a small completed subgroup; no compliance interpretation is offered [24,25] NCT: National Clinical Trial registration number

NCT number	Primary completion date (yyyy-mm-dd or yyyy-mm where the registry gives month only)	Primary completion date type	Results posted at census	Design class
NCT00842634 [46]	2013-01	Actual	No	Conventional cohort
NCT01044654 [47]	2014-12	Actual	No	Conventional cohort
NCT01252641 [48]	2014-12	Actual	No	Conventional cohort
NCT01543152 [49]	2017-07-07	Actual	Yes	Conventional cohort
NCT03081715 [50]	2018-01-23	Actual	No	Conventional cohort
NCT02225665 [51]	2018-06	Actual	Yes	Conventional cohort
NCT02793856 [52]	2018-08-20	Actual	Yes	Conventional cohort
NCT02388594 [53]	2018-09	Actual	No	Conventional cohort
NCT04213469 [54]	2021-11-01	Actual	No	Conventional cohort
NCT04560790 [55]	2022-07-05	Actual	No	Conventional cohort
NCT05812326 [56]	2022-11-16	Actual	No	Conventional cohort
NCT03432364 [57]	2022-11-17	Actual	Yes	Conventional cohort
NCT05210530 [58]	2023-01-19	Actual	No	Conventional cohort
NCT03666871 [59]	2024-01-31	Actual	No	Conventional cohort
NCT04557436 [60]	2024-03-05	Actual	No	Conventional cohort
NCT05144386 [61]	2024-11-14	Actual	No	Conventional cohort
NCT05398029 [62]	2025-02-14	Actual	No	Conventional cohort
NCT04426669 [63]	2025-05-28	Actual	No	Conventional cohort
NCT06024876 [64]	2025-07-01	Actual	No	Conventional cohort
NCT03745287 [65]	2025-07-07	Actual	Yes	Conventional cohort
NCT03653247 [66]	2025-07-17	Actual	No	Conventional cohort
NCT06065189 [36]	2025-08-12	Actual	No	Conventional cohort
NCT04601051 [67]	2025-09-12	Actual	No	Conventional cohort

Novelty

A PubMed search for a registry-based count of individualized or platform gene-editing studies returned 11 records, none of which was such a count. The closest prior work is the registry analysis of CRISPR therapeutics described in the Introduction [13]. A search for an audit of result reporting in gene editing returned no records. A broader search for design-focused reviews of gene-editing trials returned 171 records. We inspected the 15 most relevant, of which two were related to this study: the registry analysis and the scoping review of published trials [13,14]. The remaining records were not fully screened, and we make no claim about them. A search for literature linking the platform technology designation with gene therapy or gene editing returned no records.

## Discussion

Nineteen months after the first individualized CRISPR treatment, the registry contains seven individualized or platform-designated gene-editing studies among 148 eligible studies, and only two records describe a construct designed for the individual participant. Both numbers are small, and both can be verified from public fields. These counts describe the record registered on ClinicalTrials.gov at one date. They are not an estimate of how much individualized gene editing is taking place, because activity delivered outside registered studies is not captured. The counts are nevertheless small beside the prominence of individualized editing in recent commentary [9,10]. Three explanations are possible, and the registry can only partly distinguish them.

The first explanation is that individualized editing is happening but is not registered. The index case supports this directly: it was delivered under a single-patient expanded-access application, which creates no registry record [1]. If bespoke editing is delivered as clinical care rather than as research, the registry will undercount it, and its true extent cannot be known from this source. For that reason, this census cannot measure any gap between clinical practice and the registered record; it can show only that the registered record is small. Activity that leaves no public record also cannot be audited or compared at population scale. The single-patient records that do exist show what visibility looks like, including one record whose whyStopped field reports the death of the only participant, information that no publication is obliged to provide [28].

The second explanation is that the field's platform language describes the reuse of manufacturing components rather than protocol design. The products in this census are programmable, and the authors of the index case argued that shared components make rapid customization feasible [1,12]. Yet, only one record among all eligible studies registers a protocol built around designing a product for each participant [31], and one other embeds the development of a participant-specific editor inside an otherwise conventional 15-participant cohort [33]. In this set of records, platform character appears to be a property of the technology and the manufacturing process more than of the registered protocol. This may explain why a classification instrument that reads protocols finds so few platform designs.

The third explanation concerns the regulatory framework. The platform technology designation was created so that evidence about a reusable technology could carry across products, which is exactly the economic problem that individualized editing poses. However, the statute requires the platform to be already part of an approved or licensed product, and whether any product counted here relies on such a platform cannot be established from public records [15,16]. Where a bespoke editing product has no approved counterpart, this condition would limit the use of the designation for individualized editing, but we could not verify the position of any census study. No publicly verifiable designation was located for any of them, which is a statement about public evidence and not about whether a designation or another regulatory instrument exists. The lack of any public list of designations makes the program harder to study: Without a list of applications or grants, the uptake of the designation in gene editing cannot be measured from public records, and the only publicly documented grant concerns a viral vector rather than an editing technology [21,22].

The two related studies complement this one rather than compete with it. The registry analysis describes the same population in less detail and draws broader conclusions about indications and modalities [13], while the scoping review of 29 published trials describes what has been reported rather than what has been registered [14]. Neither reports duplicate classification or a reliability statistic, and our moderate kappa for design class shows why this matters: The design classes the field uses in its writing cannot be cleanly recovered from registry fields, and one reader's assignments may not be reproduced by another. The design-class counts reported here, including the census of seven, carry this uncertainty and should be read as provisional. Registry studies that use a design-class variable should routinely report kappa for it.

The practical implication is narrow and achievable. Three structured fields would make individualization visible: whether the investigational product is designed for the individual participant; whether the record is a master protocol and, if so, what the participant-level unit is; and whether the study is conducted under an expanded-access or single-patient authorization. Sponsors already know the first two at registration. The third exists as a study type, but it captures programs rather than single-patient authorizations. Without these fields, counting individualized editing studies will remain a manual task, and the field's most discussed therapeutic advance will remain the hardest to count.

Limitations

Four limitations bound every conclusion of this study: It covers one registry, design class was assigned with only moderate agreement, the second screening pass was performed by a language model, and the regulatory analysis is restricted to public sources. The findings therefore describe the record registered on ClinicalTrials.gov and not the complete universe of individualized gene-editing activity. Each limitation is set out below. This census covers one registry. Studies registered only in other national registries, studies that were never registered, and treatments delivered as clinical care under single-patient authorizations are not included. The 73 records that mention editing vocabulary outside the intervention field are excluded by design. The census is a snapshot at 17 September 2026; records are revised continuously, and the version histories we examined show how much a record can change.

The unit of analysis is the registered record, not the protocol or the study as conducted. Where a record does not state something, we report it as not stated; we did not contact sponsors and imputed no values. Design class is a judgment about protocol intent made from fields that do not record intent, which explains the moderate kappa reported above. The confidence interval for that statistic, 0.264-0.815, is wide, and the design-class counts should be read with this in mind. Fourteen of the 148 classifications were ambiguous on the evidence of the record itself, and four of the seven census records are among them (Table 2), so the census count of seven is itself sensitive to classification. The tie-break rule for overlapping classes was fixed in advance, but another defensible rule gives four platform records instead of three; we report this sensitivity analysis rather than presenting one reading as the only one possible.

The second screening pass was an independent record-by-record reading by a large language model (Claude Sonnet 5) that was instructed to decide only from the supplied fields and to quote the field its decision rested on. It is an instrument with its own types of error, not a second human reader, and the authors adjudicated its disagreements with the first pass against the record text. Readers who would weight a human second pass differently should treat the kappa values as specific to this instrument.

The result-reporting figures describe only 23 studies. We make no claim about compliance, and the Wilson interval is reported so that its width is visible.

The regulatory-route analysis is limited to public sources. Investigational applications, related correspondence, and designation requests are not public, so the absence of a public record does not show that no regulatory instrument exists for a given study; it shows only that none can be verified. We could not find released FDA correspondence for the index case and therefore describe only the route and timing stated in the primary report.

Finally, one registration discrepancy in this dataset cannot be resolved from the public record. One editing product appears with an editing mechanism in a platform trial record [37] and without any editing mechanism in its own record [38]. We kept the latter excluded rather than importing evidence from one record into another.

## Conclusions

A census of ClinicalTrials.gov on 17 September 2026 identified four single-participant or n-of-1 gene-editing studies and three multi-variant umbrella or platform-designated studies, seven in total among 148 eligible gene-editing studies. Two records describe a construct designed for the individual participant. These findings describe the registered record on ClinicalTrials.gov. They do not measure the full extent of individualized gene-editing activity, some of which, such as the index case, is delivered outside registered studies. No publicly verifiable platform technology designation was located for any census study, and the census query returned no expanded-access record. These are statements about public evidence and do not show that no regulatory instrument was used. The design-class counts rest on a classification on which two independent passes agreed only moderately (kappa, 0.540; 95% CI: 0.264-0.815), and they should be read as provisional. Individualization would need to become a registered property and not only a narrative one, before it can be counted reliably.

## Appendices

Verbatim system instruction for the second screening pass

The instruction below was supplied unchanged as the system prompt with every one of the 212 per-record requests to Claude Sonnet 5 (Anthropic, San Francisco, CA) on 17 September 2026. Each request carried only this instruction and the labelled registry fields listed in the Materials and Methods.

You screen ClinicalTrials.gov records for a study of human gene-editing therapeutics.

Decide ONLY from the record fields given. Never use outside knowledge of what a product code means unless the record itself states the mechanism; if the record does not state it, say so.

Return ONLY a JSON object, no prose, with keys: eligible: true|false; exclusion_reason: one of ["non-interventional", "laboratory assay only", "gene addition/transfer, no editing component", "withdrawn with no enrolment", "no gene-editing intervention (other)", null]; evidence_field: the field name the decision rests on; evidence_value: verbatim short quote from that field (<=200 chars); design_class: one of ["single-participant/n-of-1", "multi-variant umbrella or platform-designated", "conventional cohort", null]; design_class_evidence: verbatim short quote or field value; design_class_ambiguous: true|false; ambiguity_reason: short string or null.

Definitions

Single-participant/n-of-1 = enrolment of one participant or record describes a bespoke/patient-specific/individualized construct for one named individual. Multi-variant umbrella or platform-designated = one master protocol covering multiple genetic variants, mutations, diseases, or sub-studies or a record describing the agent as a platform/platform-designated technology. Conventional cohort = a fixed cohort receiving one defined product. Mark ambiguous when the fields support more than one class or are too sparse to choose.
